# Exploring the role of plasmapheresis prior to thyroidectomy in managing thyrotoxicosis: a comprehensive scoping review

**DOI:** 10.1007/s10047-024-01476-6

**Published:** 2024-10-10

**Authors:** Weronika Koziak, Stanisław Dudek, Zbigniew Putowski, Filippo Sanfilippo, Mateusz Zawadka

**Affiliations:** 1 2nd Department of Anesthesiology and Intensive Care, Medical University of Warsaw, Warsaw, Poland; 2 Center for Intensive Care and Perioperative Medicine, Jagiellonian University, Kraków, Poland; 3 Department of Anesthesia and Intensive Care, Policlinico-San Marco, Site “Policlinico, G. Rodolico”, Catania, Italy

**Keywords:** Plasmapheresis, Plasma exchange, Thyrotoxicosis, Thyroid crisis, Perioperative

## Abstract

A thyroid storm is the most extreme and life-threatening presentation of thyrotoxicosis. Thyroidectomy can be used for definitive treatment. It should be performed after euthyroidism is accomplished. The use of therapeutic plasma exchange (TPE) is a last resort option in cases where standard pharmacological therapy proves to be ineffective. Due to its rare prevalence, there are limited data evaluating the usefulness and efficacy of TPE as a bridging therapy to thyroidectomy. The absence of relevant literature prompted us to conduct a scoping review. The following bibliographic databases were searched for articles dated 30 November 2023: Medline, EMBASE, Web of Science and Google Scholar. The search identified 1047 records, of which 42 articles were accepted with a total of 234 patients. The dominant indications for TPE were side effects due to conventional treatment. The mean fT4 level decreased 51.9% of baseline after TPE, while the mean fT3 level decreased 66.6% of baseline. The main side effects observed with FFP were allergic reactions, while the use of an albumin solution was associated with perioperative bleeding. Based on the limited data available in the literature, we recognize plasmapheresis as an effective treatment option for reducing thyroid hormone levels prior to thyroidectomy in patients with thyrotoxicosis. Available data suggest that it might be reasonable to limit the number of sessions in favor of an earlier surgical intervention. To reduce the risk of bleeding, FFP may be a better option as a replacement fluid, especially in the session prior to thyroidectomy.

**Supplementary Information:**

The online version contains supplementary material available at 10.1007/s10047-024-01476-6.

## Introduction

Hyperthyroidism refers to an elevated level of thyroid hormones due to increased synthesis and secretion by the thyroid gland, whereas thyrotoxicosis refers to a clinical condition resulting from the action of inappropriately high levels of circulating thyroid hormones. The most extreme and life-threatening presentation of thyrotoxicosis is the thyroid storm. This condition manifests as rapid clinical worsening and multiple-organ decompensation. Thyroid storm carries a high rate of mortality, estimated at 8 to 25%, and require rapid aggressive therapy [1–4].

According to the current 2016 guidelines, both the American Thyroid Association (ATA) and Japan Thyroid Association and Japan Endocrine Society (JTA) recommend comprehensive management of thyroid storm that includes the use of antithyroid drugs (ATDs), beta-blockers, inorganic iodine, corticosteroids, and body temperature reduction [1, 4].

Either radioactive iodine therapy or thyroidectomy can be used for the definitive treatment of thyrotoxicosis. However, it is also recommended that surgery is performed after euthyroidism is accomplished [5]. The optimization of patients with thyrotoxicosis before surgery is very important. Indeed, the release of thyroid hormones can cause several side effects, the most important of which are probably at the cardiovascular level. Tachycardia and sympathetic hyperactivity may put patients at increased risk of complications. In the setting of sepsis, both tachycardia and hyperdynamic left ventricular systolic function have been associated with mortality [6, 7], highlighting the importance of modulating sympathetic drive. In some cases, though, standard therapy of thyrotoxicosis proves to be ineffective or cannot be used due to its side effects, making it necessary to use alternative options [4]. Among these, a last resort for treating thyrotoxicosis is represented by the use of therapeutic plasma exchange (TPE), which has been shown to be effective at lowering patients’ hormone levels and achieving the euthyroidism condition required for scheduling surgery [4, 8, 9].

In thyrotoxicosis, TPE allows for a rapid decrease in circulating thyroid hormone levels, as a large proportion of these hormones are bound to proteins. Interestingly, treatment with TPE also reduces blood levels of drugs that may be responsible for some cases of thyrotoxicosis, such as amiodarone, which has a half-life of up to several months when undergoing chronic therapy [8, 9].

Due to its rare prevalence, there are no randomized studies in the literature evaluating the usefulness and efficacy of TPE for the treatment of thyroid storm [4]. The absence of literature addressing the use of TPE for the treatment of thyrotoxicosis as a bridging therapy to thyroidectomy has prompted us to conduct a scoping review.

## Methods

### Searches

To identify potentially relevant documents, the following bibliographic databases were searched for articles dated 30 November 2023: Medline, EMBASE, Web of Science and Google Scholar. The keywords were organized in two groups, one to identify the treatment and one for the condition/disease. The following search string was applied: (plasmapheresis OR plasma exchange OR apheresis) AND (thyroid storm OR thyroid crisis OR thyrotoxicosis OR thyrotoxic crisis OR thyrotoxic storm). No search limitations and filters were applied.

### Data extraction (selection and coding)

Studies were eligible for inclusion if they concerned patients with thyrotoxicosis who underwent TPE prior to thyroidectomy. No control group was included. Due to the scoping nature of the review, we did not set out specific primary or secondary outcomes.

Titles and abstracts were screened for studies meeting the inclusion criteria. A full-text review was performed for studies meeting the inclusion criteria in the English language.

The inclusion criteria included the use of plasmapheresis in patients prior to surgery to reduce thyroid hormone levels and the inclusion of information on the plasmapheresis procedure performed. These criteria are summarized in Table 1 according to the PICOS framework.

**Table 1:** PICOS inclusion and exclusion criteria

Parameters	Inclusion criteria
Population	Patients with thyrotoxicosis qualified to definitive treatment
Intervention/comparator	Preoperative plasmapheresis
Outcomes	Information about the patient, such as age, gender, etiology of thyrotoxicosis; indications for preoperative plasmapheresis, treatment protocol used, number and frequency of plasmapheresis sessions, type of replacement fluid, volume and flow rate, time from the last session to surgery, thyroid hormone levels before and after plasmapheresis, side effects of plasmapheresis, perioperative complications, and survival
Study design	Randomized studies, cohort studies, case series and case reports, systematic reviews, guidelines, conference reports

The following data were extracted from each study: information about the patient(s), such as age, gender, etiology of thyrotoxicosis, indications for preoperative plasmapheresis, treatment protocol used, number and frequency of plasmapheresis sessions, type of replacement fluid, volume and flow rate, time from the last session to surgery, thyroid hormone levels before and after plasmapheresis, side effects of plasmapheresis, perioperative complications and survival.

## Results

### Data extraction

In total, 1037 records were identified: 191 results were obtained via Medline, 453 from the EMBASE database, 193 from the Web of Science database and 200 from the Google Scholar database, which then underwent screening (Fig. 1 in the Supplement). Finally, 42 papers were included in this review [3, 10–51].

### Patients’ characteristics

A total of 234 patients from 42 papers were included in this review. The patients had a pooled mean age of 42.8 ± 11.4 years and were predominantly females (n = 144, 61.5%).

The most frequent etiology of thyrotoxicosis was Graves’ disease, which represented over two thirds of the reported cases (n = 163, 69.7%). A complete list concerning the etiology in the full cohort of included series/reports is provided in Table 2.

**Table 2:** Patient data and averages (n = 234)

Variables	Findings
Female (n, %)	144 (61.5)
Age (years) (mean ± sd)	42.8 ± 11.5
Etiology of hyperthyroidism (n, %)	
Graves’ disease	163 (69.7)
Amiodarone-induced thyrotoxicosis	27 (11.5)
Toxic multinodular goiter	32 (13.7)
Toxic adenoma	3 (1.3)
Familial non-autoimmune autosomal dominant hyperthyroidism	1 (0.4)
Jod-Basedow syndrome	2 (0.9)
Amiodarone-induced thyrotoxicosis with underlying Graves’ disease	1 (0.4)
Thyroiditis	2 (0.9)
Etiology unknown	3 (1.3)

### Plasmapheresis: indications

The dominant indications for TPE were side effects due to conventional treatment (n = 91, 39.1%), such as hepatotoxicity (n = 36, 15.4%), agranulocytosis (n = 26, 11.1%), allergic reactions (n = 4, 1.7%), thrombocytopenia (n = 1, 0.4%), unspecified (n = 24, 10.3%). The other indications are summarized in Table 3.

**Table 3:** Indications for TPE

Indication	Number of patients (%)
Severe adverse effects due to conventional treatment	91 (39.1%)
Hepatotoxicity	36 (15.4%)
Agranulocytosis	26 (11.1%)
Allergic reactions	4 (1.7%)
Thrombocytopenia	1 (0.4%)
Unspecified	24 (10.3%)
Thyroid storm refractory to conventional treatment	69 (29.6%)
Emergency (including severe clinical condition, thyrotoxicosis prior to emergency surgery for another reason)	36 (15.5%)
Contraindications to conventional treatment	24 (10.3%)
Undefined	13 (5.6%)

### The choice of method and replacement fluid

Centrifugal therapeutic plasma exchange (cTPE) was used in 185 patients (93.4%) and membrane therapeutic plasma exchange (mTPE) in 13 patients (6.6%). In 11 patients, both the cTPE and mTPE were used during treatment [25]. 25 patients could not be assigned to either method due to lack of data [3, 16, 17, 20, 25, 26, 29, 35, 37, 39–45, 47, 50, 51].

Fresh frozen plasma (FFP) was used in 101 patients (43.2%), an albumin solution in 46 (19.7%), a combination of both FFP and albumin in 47 (20.0%), and hydroxyethyl starch in 3 patients (1.3%). In 37 instances (15.8%), no information was provided about the selected replacement fluid. We found no differences in the effectiveness of TPE in lowering thyroid hormone levels according to the fluid used.

### Volume and frequency

The volume used during plasmapheresis was calculated as 1–1.5 × of patient’s plasma estimated volume in 7 studies [8, 10, 12, 16, 19, 28, 42]. In the remaining cases containing this information, the plasma volume ranged from 1.5 to 4 L [11, 17, 22, 25, 30–32, 34, 36, 47]. A total of 83 patients underwent TPE sessions every 24–48 h [12, 16, 19, 21, 22, 27, 32, 39, 41, 44]. TPE sessions performed within a shorter time window (every 7–19 h) were described in 2 case reports [11, 17].

### Flow rate and duration of session

In the majority of the series/reports, the flow rate ranged from 50 to 80 ml/min [8, 16, 19, 22, 44]. One trial used a higher flow rate of 200 ml/min [30]. When specified, the duration of a TPE cycle ranged from 2 to 5 h [8, 16, 19, 29, 32, 33, 47].

### The time between the last plasmapheresis session and surgery

Surgical intervention was performed within 24 h after the last TPE session in 31 patients. Significant perioperative bleeding was reported in 6 patients [10, 15, 17–19, 22, 24, 28, 34, 36, 38, 42, 46]. Surgery was performed after 48 h in 5 patients [22, 31, 42, 45] and after 72 h in 1 patient [30]. In these reports/series, we found no information on perioperative bleeding in either group. For 197 patients, no information was provided on the time interval between the last TPE session and surgery.

### Effects of therapeutic plasmapheresis on thyroid hormone levels

**Fig. 1:**
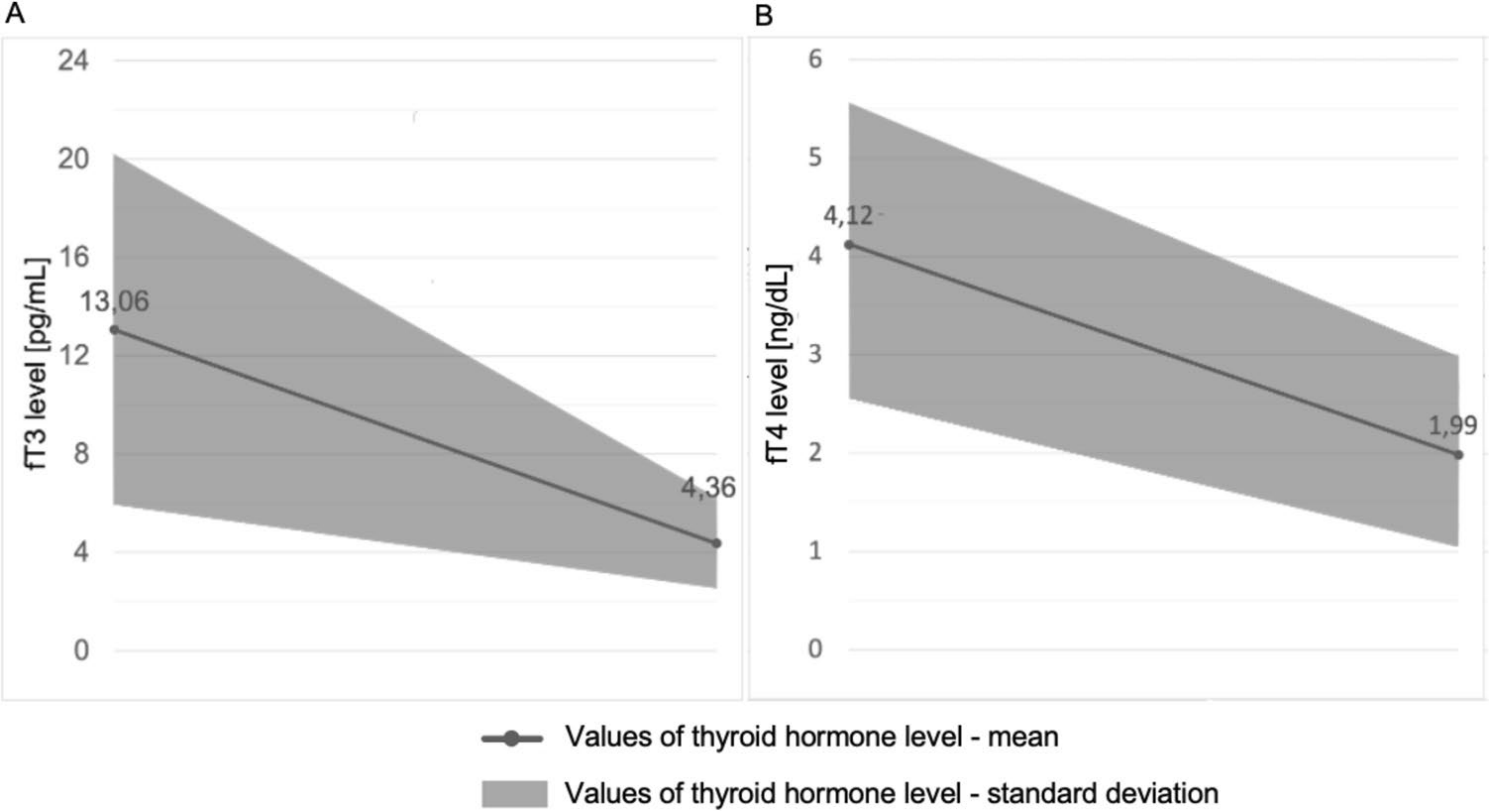
Mean values of thyroid hormone levels (fT3, fT4) pre- and post-TPE

The evaluation of the reduction in thyroxine-free (fT4) hormone levels was possible in 225 patients. Based on our analysis of pooled data, the average level of the fT4 hormone was halved by TPE, with a reduction from 4.12 ± 1.48 ng/dL to 1.99 ± 0.99 ng/dL (51.9% reduction in the baseline value; Fig. 1B).

The evaluation of the decrease in the fT4 hormone levels in relation to the number of sessions was possible for 225 patients. The average decrease in hormone levels was 0.544 ng/dL per session. This represents an average 13.0% decrease per session compared to the pre-TPE value.

The reduction in free triiodothyronine (fT3) hormone levels could be assessed in 178 patients. Our analysis showed that the mean level of the fT3 hormone decreased from 13.06 ± 7.15 pg/mL to 4.36 ± 2.28 pg/mL (66.6% of the baseline level; Fig. 1A).

In 177 patients, it was possible to evaluate the decrease in the fT3 hormone levels in relation to the number of sessions. The average reduction in hormone levels was 2.31 pg/mL per session. This represents an average decrease of 16.3% per session compared to pre-TPE levels.

The mean number of plasmapheresis sessions among the 232 patients was 4.74 ± 2.4.

**Fig. 2:**
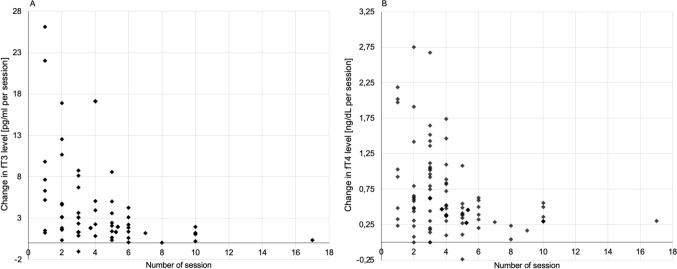
Change in the fT3 and fT4 level per session

A decrease in the fT3 level was inversely correlated with the number of sessions used. In 56 patients, the number of sessions was less than or equal to 3, with an average decrease in fT3 levels of 3.8 ± 5.2 pg/ml (26.6 ± 18.8%) per session, compared to 121 patients, in whom the decrease was 2.8 ± 4.2 pg/ml (11.6 ± 3.5%) per session when the number of sessions was greater than 3 (Fig. 2A). A similar correlation between the level of fT4 hormone and the number of sessions was not found (Fig. 2B).

Out of 234 patients, only 4 (1.7%) patients did not respond to TPE, with a very marginal decrease in fT3 and fT4 levels [26, 32, 37].

### Adverse effects

Side effects were reported in 44 patients (18.8%).

Mild allergic reactions occurred in 14 patients (6.0%). In all the patients, FFP was used as a replacement fluid (in 12 patients, FFP was used as the sole replacement solution, and in the remaining 2 patients, FFP was combined with 5% albumin) [16, 20, 21, 25, 36, 44].

Perioperative bleeding was described in 8 patients (3.4%), 3 of whom required blood transfusion. 5% albumin was used as a replacement fluid in 5 patients, hydroxyethyl starch was used in 1 patient, and adequate data were not available for the remaining 2 patients [19, 20, 34, 36, 42].

Catheter-related infections were reported in 3 patients (1.3%). Apheresis-catheter-related upper extremity deep vein thrombosis occurred in 1 patient (0.4%) [8, 10, 46].

Transient hypocalcemia was observed in 13 patients (5.6%), and persistent hypocalcemia in one patient (0.4%). In one patient (0.4%), the TPE procedure was discontinued because of a mild decrease in blood pressure (drop to 90/60 mmHg). 4 patients (1.7%) failed to respond to plasmapheresis (no reduction in thyroid hormone levels). However, based on the available information, we could not determine what replacement fluid was used in these patients [15, 19, 20, 25, 36, 40].

### Survival rate

Of the 234 patients, 4 (1.7%) died during hospitalization—2 due to hospital-acquired infection, one as a result of pulmonary embolism, and one for severe thyrotoxicosis resistant to treatment [26, 32].

### Impact on the outcome of surgery

Good results were achieved in all patients who underwent surgery. In the eight cases mentioned above, there was post-operative bleeding, and blood transfusion was required in three patients [19, 20, 34, 36, 42]. All patients who underwent thyroid surgery after TPE survived.

## Discussion

TPE appears to be an effective method for achieving euthyroidism in patients with thyrotoxicosis before thyroidectomy. Due to the lack of randomized controlled trials on this topic, the available evidence is of a low quality, and this aspect is clearly reflected in the guidelines where TPE receives grade 2C recommendation) [7].

Among the existing guidelines only the American Society for Apheresis (ASFA) includes the use of plasmapheresis as a method of preparing a patient with thyrotoxicosis for thyroidectomy [9].

In the retrieved studies, TPE reduced basal thyroid hormone levels by more than 50%, which indicates a good efficacy, especially considering that in a significant proportion of these patients, the conventional treatment had failed.

There are currently two main replacement fluids used for TPE: FFP and/or albumin. The advantage of FFP is it increases the concentration of thyroxine-binding globulin, which in turn binds specifically to thyroxine and triiodothyronine, further reducing their free plasma concentrations; nonetheless, albumin also decreases free thyroid hormone levels [9]. There are currently no randomized trials available showing an advantage for either of these fluids. The ASFA guidelines state that both fluids can be used, but there is no preference for one over the other [7]. In contrast, the guidelines of the JTA recommend the use of FFPs based on their preference in numerous case reports and the expected greater decrease in hormone levels [4].

In our analysis, we did not find any differences in the effectiveness of lowering hormone levels between these two types of replacement fluid. However, it should be noted that only patients treated with FFP experienced allergic reactions during TPE. Conversely, when comparing albumin solution to FFP, bleeding seemed to be reported only in patients in whom an albumin solution was used. Koh et al. suggested the use of FFP in the final session to reduce intraoperative bleeding due to the increase in coagulation factors provided by the reinfusion of FFP; this strategy was used in four patients included in our analysis [12, 15, 17, 42]. In one of these four patients, significant intraoperative bleeding occurred, requiring transfusion of two units of blood.

The ASFA recommends the use of 1–1.5 total plasma volume, performed daily to every 3 days, depending on the patient's clinical condition [7]. Müller et al. recommended daily plasmapheresis with 40–50 ml/kg replacement fluid until clinical improvement [6]. However, data on the above technical aspects of plasmapheresis are limited. Based on the available data, the majority of the authors followed the established guidelines, with only two papers reporting a frequency of sessions (7–19 h between sessions) higher than the recommended frequency [11, 17]. One study also described the use of a much higher flow rate than recommended (200 ml/min) [30].

According to ASFA guidelines, TPE should be continued until clinical improvement is achieved, which is usually achieved after 3–6 sessions. This finding is consistent with the results of our analysis, where the average number of sessions was 4.74. There are no data in the literature regarding the target hormone levels achieved prior to surgery, but efforts should be made to normalize their levels. According to our analysis, the average fT3 level at which the decision to operate was made was 4.36 pg/ml, and the average fT4 level was 1.99 ng/dL.

It may be worth highlighting that the effectiveness of lowering thyroid hormone levels per session decreased as the number of sessions increased. This may indicate the appropriateness of limiting the number of sessions and deciding not to continue TPE in favor of earlier surgical intervention.

There is no consensus on how long after the last plasmapheresis session, thyroidectomy should be performed. One consideration is the increased risk of perioperative bleeding after plasmapheresis using an albumin solution as a replacement fluid due to plasma substitution with a solution without coagulation factors. Ozbey et al. recommended postponing surgery for up to 48 h and using FFP as a replacement fluid in the last plasmapheresis session to reduce this risk. On the other hand, the effect of plasmapheresis is mostly transient, and thyroid hormone levels usually rise again the next day [7]. Unfortunately, the time between the final plasmapheresis session and surgery has rarely been reported in the literature. Due to the lack of data, the relationship between the incidence of intraoperative bleeding and the short time between the last plasmapheresis session and surgery, as noted by Ozbey et al., remains unclear. Notably, intraoperative bleeding occurred only in patients who underwent surgery within 24 h after the last session, and no cases were reported in patients in whom this interval was extended beyond 24 h.

However results from this scoping review should be interpreted with caution. It should be taken into consideration that this may be due to publication bias, as TPE-unresponsive patients may not have been reported in the literature. Additionally, the low overall mortality rate in our study (below 2%) may be affected by publication bias.

## Conclusions

Based on the limited data available in the literature, we recognize plasmapheresis as an effective treatment option for reducing thyroid hormone levels prior to surgery in patients with thyrotoxicosis. Available data suggest that it might be reasonable to limit the number of sessions in favor of an earlier surgical intervention. To reduce the risk of bleeding, FFP may be a better option as a replacement fluid, especially in the session prior to surgery.

## Supplementary Information

Below is the link to the electronic supplementary material.

Supplementary file1 (PDF 142 KB)

## Abbreviations

ASFA: American Society for Apheresis
ATA: American Thyroid Association
ATDs: Antithyroid drugs
ECMO: Extracorporeal Membrane Oxygenation
FFP: Fresh frozen plasma
fT3: Free triiodothyronine hormone
fT4: Thyroxine-free hormone
JTA: Japan Thyroid Association and Japan Endocrine Society
PICOS: Population, Intervention, Comparison, Outcomes and Study
TPE: Therapeutic plasma exchange
