# Supplementary figures and images for: Exploring the role of plasmapheresis prior to thyroidectomy in managing thyrotoxicosis: a comprehensive scoping review
Source: J Artif Organs. 2024 Oct 10;28(2):146–53. doi: 10.1007/s10047-024-01476-6 (PMC12078344; doi:10.1007/s10047-024-01476-6)

**Figure 1.** Flow-chart: Selection of eligible studies

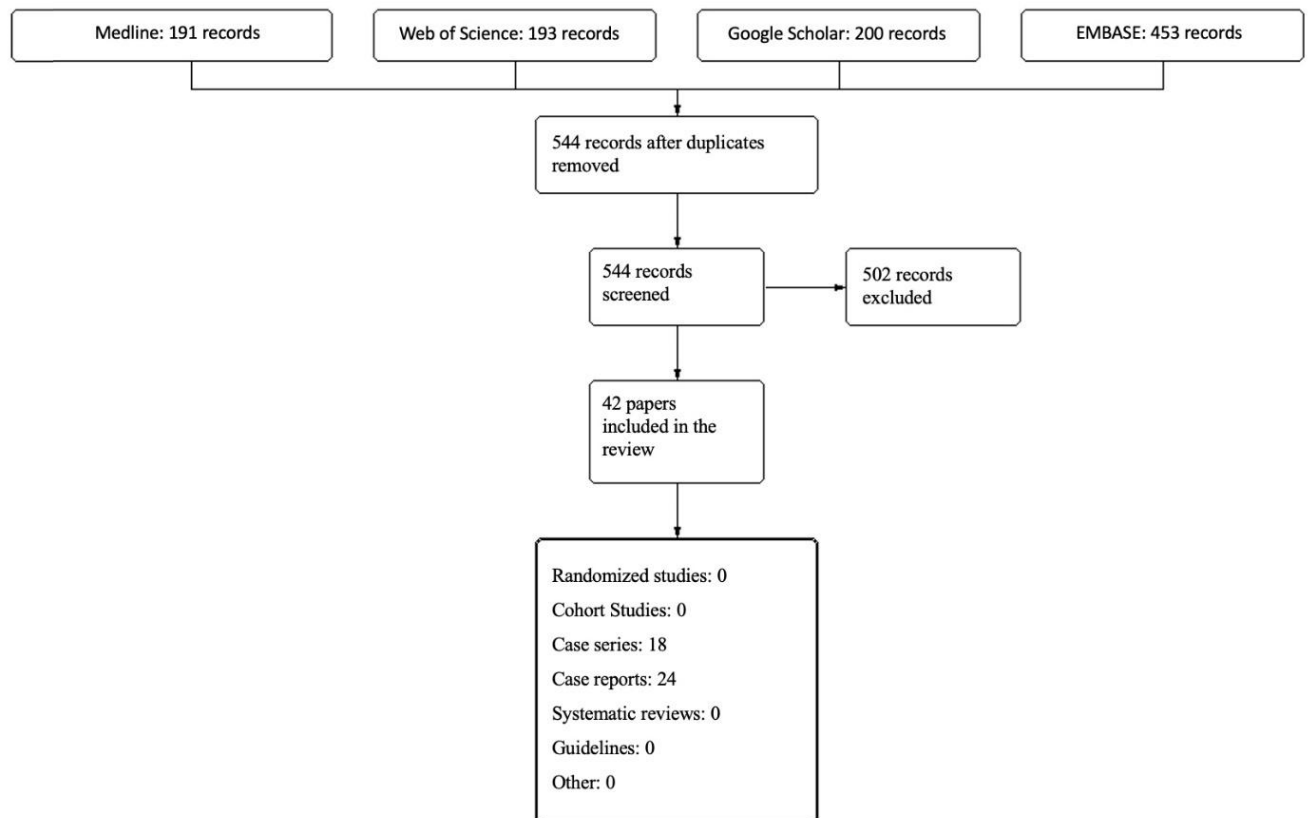

Supplement: Supplementary file 1 — Supplementary file1 (PDF 142 KB) [file 10047_2024_1476_MOESM1_ESM.pdf]
